# Using cultured canine cardiac slices to model the autophagic flux with doxorubicin

**DOI:** 10.1371/journal.pone.0282859

**Published:** 2023-03-16

**Authors:** Asma Boukhalfa, Sally R. Robinson, Dawn M. Meola, Nicholas A. Robinson, Lauren A. Ling, Joey N. LaMastro, Jenica N. Upshaw, Lakshmi Pulakat, Iris Z. Jaffe, Cheryl A. London, Howard H. Chen, Vicky K. Yang

**Affiliations:** 1 Molecular Cardiology Research Institute, Tufts Medical Center, Boston, Massachusetts, United States of America; 2 Cummings School of Veterinary Medicine, Tufts University, North Grafton, Massachusetts, United States of America; 3 Tufts University School of Medicine, Boston, Massachusetts, United States of America; 4 Division of Cardiology, Tufts Medical Center, Boston, Massachusetts, United States of America; Faculty of Medicine, University of Belgrade, SERBIA

## Abstract

Chemotherapy-induced impairment of autophagy is implicated in cardiac toxicity induced by anti-cancer drugs. Imperfect translation from rodent models and lack of *in vitro* models of toxicity has limited investigation of autophagic flux dysregulation, preventing design of novel cardioprotective strategies based on autophagy control. Development of an adult heart tissue culture technique from a translational model will improve investigation of cardiac toxicity. We aimed to optimize a canine cardiac slice culture system for exploration of cancer therapy impact on intact cardiac tissue, creating a translatable model that maintains autophagy in culture and is amenable to autophagy modulation. Canine cardiac tissue slices (350 μm) were generated from left ventricular free wall collected from euthanized client-owned dogs (n = 7) free of cardiovascular disease at the Foster Hospital for Small Animals at Tufts University. Cell viability and apoptosis were quantified with MTT assay and TUNEL staining. Cardiac slices were challenged with doxorubicin and an autophagy activator (rapamycin) or inhibitor (chloroquine). Autophagic flux components (LC3, p62) were quantified by western blot. Cardiac slices retained high cell viability for >7 days in culture and basal levels of autophagic markers remained unchanged. Doxorubicin treatment resulted in perturbation of the autophagic flux and cell death, while rapamycin co-treatment restored normal autophagic flux and maintained cell survival. We developed an adult canine cardiac slice culture system appropriate for studying the effects of autophagic flux that may be applicable to drug toxicity evaluations.

## Introduction

With profound advances in cancer survivorship, cardiovascular disease has become the leading cause of non-cancer-related morbidity and mortality in cancer patients following treatment [1–3]. Traditional chemotherapies such as anthracyclines (*e*.*g*., doxorubicin), as well as many novel therapies such as small molecule kinase inhibitors, increase the risk of heart failure in cancer patients. In a pooled analysis in the metastatic cancer setting, reductions in left ventricular ejection fraction or clinical heart failure occurred in a dose-dependent manner from 6% of those treated with 250 mg/m^2^ up to 76% of patients at a cumulative doxorubicin dose of 600 mg/m^2^ [4]. Even at lower cumulative doses of doxorubicin commonly used in breast cancer (240 mg/m^2^), 10–15% of patients develop cardiomyopathy and >5% develop heart failure [5, 6]. Newer targeted agents and combination therapies are generally considered safer, but evidence now suggests that many of these have potential for cardiotoxicity through mechanisms not yet clearly defined [7]. Furthermore, there are currently no routinely used cardioprotective strategies to prevent toxicity, in part due to gaps in knowledge regarding mechanistic drivers [1, 8]. Like humans, dogs develop cancer spontaneously with over 4 million dogs diagnosed annually [9]. Importantly, dogs develop similar cardiotoxicity from treatment of their cancer. For example, doxorubicin-induced cardiotoxicity can occur in dogs who have received a total cumulative dose as low as 90 mg/m^2^ [10]. As with humans, the lack of cardioprotective strategies has hampered treatment advances.

One potential cause of chemotherapy-induced cardiotoxicity is dysregulated autophagy, resulting in cardiomyocyte cell death [11, 12]. Autophagy is activated in response to a myriad of environmental and cellular stresses, and is manifested by sequestration of parts of the cytoplasm to form double-membraned vesicles (autophagosomes) that are subsequently trafficked to the lysosomes for degradation. The degraded cellular material is subsequently recycled, used as nutrients, or exocytosed. Disruption of this multi-step process can lead to dysregulation of the autophagic flux, a dynamic measure of the degradative capacity of autophagy, followed by cell death rather than cell repair. Individual steps in autophagic flux can be influenced by both intrinsic regulators (for example: mTOR signaling, reactive oxygen species) and extrinsic regulators (drugs). The integrity of the autophagic pathway can be assessed by quantifying autophagic markers including lipidated Light Chain 3 (LC3-II) and autophagic cargo adaptor p62 [13, 14]. Specifically, LC3-II formation from LC3-I indicates the successful formation of autophagosomes, and p62 marks the trafficking of intracellular contents to the autophagosomes [15]. Previous preclinical *in vitro* and *in vivo* (murine) studies linked impaired autophagy to doxorubicin-induced cardiotoxicity and further demonstrated that restoration of autophagic flux can be achieved by pharmacological, physiological, or genetic means to improve cardiomyocyte salvage and overall cardiac function [16–19].

Successful clinical translation of agents that restore autophagic flux has been challenging, in part due to a lack of credentialed large animal model systems that accurately recapitulate the process of autophagy following cardiac insult. While such processes can be evaluated *in vitro*, there are no readily available stable culture systems for mature cardiac tissues that faithfully model human cardiac responses. Rodent-derived cardiomyocytes are most often generated from neonates and lack the typical ion channel and myofilament composition found in human cardiomyocytes [20]. Human iPSCs-derived cardiomyocytes can be used in place of those from rodents, but the resultant cardiomyocytes often lack adult cell phenotypes [21, 22], and these immature cells have different ion handling behavior [23]. Furthermore, studies employing individual cardiomyocytes, either through enzymatic digestion or differentiation from iPSCs ignore critical interactions with other cells in the heart including endothelial cells, fibroblasts, and immune cells. Given that many of the newer anti-cancer drugs may induce cardiotoxicity by targeting key support cells within cardiac tissues, it is critical that an *in vitro* model system include these elements. Consequently, we sought to credential a novel platform using cardiac slices, a format in which the natural cardiac microstructure is preserved, to study how cancer treatments influence autophagic flux [20].

For the following studies, we chose to focus on heart tissue derived from dogs. Canine cardiomyocytes are more similar in ion channel behavior to human cardiomyocytes than those of rodents [24, 25]. Furthermore, dogs develop spontaneous cancers and similar to humans, receive a variety of chemotherapy and small molecule treatments [26]. Indeed, canine cancer patients treated with cumulative doxorubicin doses of >180–240 mg/mm^2^ develop similar signs of cardiotoxicity including development of life threatening heart failure [10]. Finally, precision-cut tissue slices derived from canine cancer patients can be readily obtained and used in *ex vivo* studies. As such, the purpose of this study was to demonstrate that cardiac tissue derived from adult canines can be successfully cultured as intact slices and that this novel platform can be readily employed to evaluate changes in autophagic flux following exposure to anti-cancer agents.

## Methods

### Canine cardiac slice culture

Adult canine hearts were collected from client-owned dogs whose owners elected euthanasia at the Tufts University Foster Hospital for Small Animals with owner consented through the tissue donation program at Tufts University Cummings School of Veterinary Medicine. Tissue collected from the client-owned patient donation program has received Tufts Institutional Animal Care and Use Committee (IACUC) exemption and does not require IACUC review and approval. Hearts were collected from dogs without known cardiac disease and who have not received any cancer treatment. The demographic information of these dogs is shown in Table 1. Humane euthanasia was performed by the attending veterinarian according to standard protocols in veterinary hospitals, and each heart was harvested within 10 minutes of euthanasia and kept in cold (4°C) sterile cardioplegic solution (5.5 mM glucose, 0.5 mM MgSO_4_, 24 mM KCl, 15 mM NaHCO_3_, 109 mM NaCl, 0.9 mM NaH_2_PO_4_, 1.8 mM CaCl_2_, adjusted to pH of 7.4) for 24 hours. Cardiac slicing protocol was modified from previous report [27]. Briefly, trans-mural sections of the LV free wall measuring 1 cm × 1 cm were excised. These tissue blocks were then affixed to the cutting block of a vibrating microtome (Campden Model 5100mz) using cyanoacrylate glue with the epicardial surface facing down, and then submerged in cold oxygenated slicing solution (30 mM 2,3-butanedione monoxime, 1 mM glucose, 10 mM HEPES, 6 mM KCl, 140 mM NaCl, 1 mM MgCl_2_, 1.8 mM CaCl_2_, adjusted to pH of 7.4). The top ¼ section of the tissue at the endocardial surface was first sliced and removed to create a flat cutting surface. Then, slices of 350-μm thickness were cut with a frequency of 75 Hz and amplitude of 1.5 mm, advancing at 0.1 mm/s. Resulting slices were washed once in culture media (M199 with 0.1% BSA, 1× ITS, 10 mM 2,3-butanedione monoxime, 1× chemically defined lipid, 100 U/ml penicillin/streptomycin, 1× HEPES) before being placed on a 0.3 μm pore size transwell surface of a 6-well transwell culture plate at the air-media interface at 37°C with 5% CO_2_. Slices were subsequently treated with doxorubicin (Cayman Chemical, 15007), rapamycin (Cayman Chemical, 13346), or chloroquine (Cayman Chemical, 14194).

**Table 1:** Demographic information of dogs used in this study.

Breed	Age (Years)	Sex	Weight (kg)
West highland terrier	14	Castrated male	15
English Springer Spaniel	7	Spayed female	17
Boxer	10	Female	26
Dachshund	6	Castrated male	10
Mixed breed	13	Spayed female	25
German wirehaired pointer	9	Castrated male	25
Yorkshire terrier	12	Spayed female	4

### Mouse cardiac slice culture

The study complied with all institutional and national requirements for the care and use of laboratory animals (approved by Tufts IACUC, protocol number G2022-122). Adult B6 (Jackson Laboratory) male mice, a strain of mice with normal hearts, were euthanized using CO_2_, and each heart was harvested immediately after euthanasia and kept in cold (4°C) sterile slicing solution (as described above) for transport and immediate slicing. The base of the heart was transected to remove the great vessels and atria and create a flat surface perpendicular to the long axis of the heart. This surface was then affixed to the cutting block of a vibrating microtome using cyanoacrylate glue and submerged in cold oxygenated slicing solution. 300-μm slices were cut with a frequency of 75 Hz and amplitude of 1.5 mm, advancing at 0.1 mm/s. Resulting slices were washed once in Claycomb media with 100 μM norepinephrine, 10% fetal bovine serum, and 4 mM L-glutamine, before being placed on a 0.3 μm pore size transwell in the same Claycomb media (or the canine media above) at the air-media interface at 37°C with 2% CO_2_. A flow chart summarizes the experiments performed with the cardiac slices is shown in S1 Fig.

### Cardiac slice MTT assay

Cell viability within the slices was assessed using a 3-(4,5-dimethylthiazol-2-yl)-2,5-diphenyltetrazolium bromide (MTT) assay (Invitrogen, M6494). First, the slices were incubated in 1 ml of 0.5 mg/ml MTT solution at 37°C with 5% CO_2_ (canine) or 2% CO_2_ (mice) for 1 hour. The slices were then placed in 1 ml of PBS for 5 minutes at 37°C with 5% CO_2_ (canine) or 2% CO_2_ (mice), and subsequently incubated in DMSO for 30 minutes at 37°C with 5% CO_2_ (canine) or 2% CO_2_ (mice). The absorbance of the resulting supernatant was assessed by Optical Density measured at 555 nm and normalized by the weight.

### Cardiac slice histology

Slices were fixed in 10% formaldehyde in PBS for 24 hours, then in 70% ethanol prior to sectioning. Hematoxylin and eosin (H&E) staining and terminal deoxynucleotidyl transferase dUTP nick end labeling (TUNEL) (Abcam, ab206386) were then performed. Quantification of the number of TUNEL+ nuclei was assessed by examining at least five light microscopy images from independent biological samples by treatment-blinded investigators. Since we were unable to validate any antibodies to caspase 3 for accurate quantification of canine protein, TUNEL staining was used to quantify apoptosis for the canine cardiac slices.

We used the Bellingham grading system to describe severity of pathologic changes in heart tissue secondary to doxorubicin treatment as it was developed to characterize tissue damage specifically in the context of doxorubicin cardiotoxicity [28]. Grade 0 indicates no change from normal myofibrillar structure, grade 1 indicates early myofibrillar loss, grade 2 indicates marked myofibrillar loss with cytoplasmic vacuolization, and grade 3 indicates diffuse cell damage with marked changes.

### Tissue fluorescence imaging

Direct imaging of the inherent doxorubicin fluorescence was carried using an IVIS spectrum (Perkin Elmer). After exposure to 0–50 μM of doxorubicin for 24 hours, the slices were washed with PBS overnight before imaging. Imaging was performed with the filter setting 500 nm excitation/540 nm emission, 20 second exposure, with 135 μm in-plane resolution. Doxorubicin fluorescence signal was quantified in ImageJ (NIH) by calculating the signal-to-noise ratio (mean fluorescence intensity of heart/standard deviation of air).

### Protein immunoblotting

Cardiac slices were flash-frozen in liquid nitrogen, sonicated, and homogenized on ice in radio-immunoprecipitation assay buffer supplemented with protease inhibitors (Boston BioProducts) per manufacturer’s instructions. Proteins were extracted by constant agitation for 30 minutes before centrifugation. Protein concentration was measured by Bradford assay (BioRad), and 20–40 μg of total protein was loaded for electrophoresis and transferred to the polyvinylidene difluoride membrane. The membrane was processed for immunoblotting against LC3 (CST 3868, rabbit monoclonal, clone: D11; 1:1000, antibody ID: AB_2797680), p62 (CST 5114, rabbit polyclonal, 1:1000, antibody ID: AB_10624872), and actin (Sigma A5316, mouse monoclonal, clone: AC-74; 1:5000, antibody ID: AB_476743) and imaged with a ChemiDoc XRS molecular imager (BioRad). Immunoblot images are analyzed using ImageJ by measuring the LC3-I, LC3-II, p62, and actin signal intensity and calculating the LC3-II to LC3-I, LC3-II to actin, and p62 to actin ratios.

### Immunostaining and confocal microscopy

Cardiac slices were fixed, sectioned, permeabilized, and stained with the LC3 antibody (MBL M152-3, mouse monoclonal, 1:100, antibody ID: AB_1279144) followed by fluorescent anti-mouse secondary antibodies (Jackson ImmunoResearch Lab 715-585-151, donkey polyclonal, 1:200, antibody ID: AB_2340855). After nuclear staining with 4’,6-diamidino-2-phenylindole (DAPI), the sections were visualized on a Falcon SP8 system (Leica) with images acquired at 1024×1024 resolution. The images were analyzed in ImageJ.

### Statistical analysis

Normality of data distribution was assessed using the D’Agostino-Pearson normality test. For normally distributed data, statistical analysis was performed using the one-way ANOVA test with Tukey post-test for comparison of three or more groups or Dunnett’s test for multiple comparison test against a single baseline. All statistical analysis and plots of mean ± SEM were performed using Prism (GraphPad). *p<0.05; **p<0.01; ***p<0.001; ****p<0.0001.

## Results

### Canine cardiac slices maintain viability in culture longer than mouse cardiac slices

**Fig 1:**
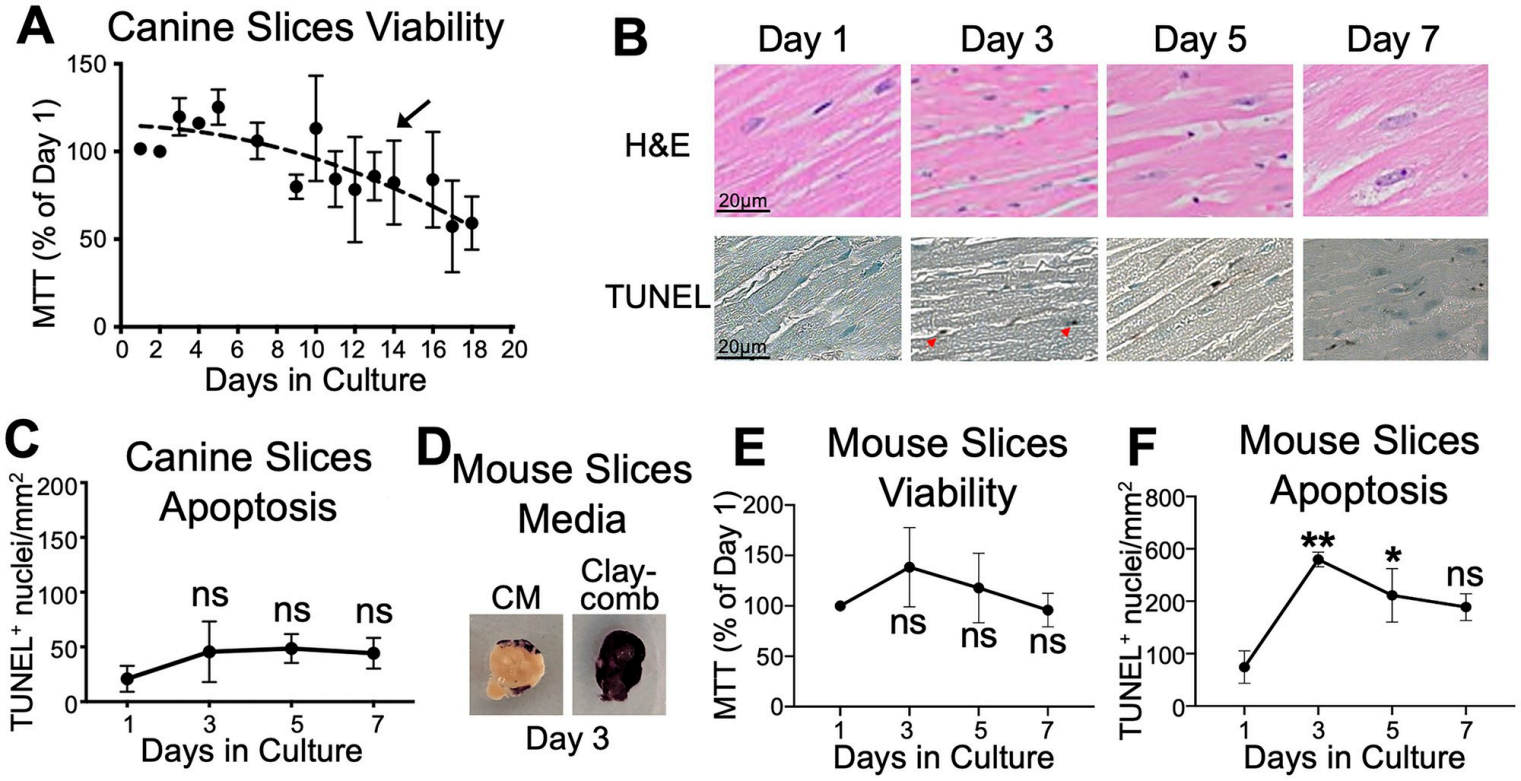
Canine cardiac slices are viable for >7 days in culture. A. No significant reduction in adult canine cardiac slice viability through 14 days of culturing (arrow) by MTT staining was noted. N = 7 hearts. B. H&E staining demonstrated minimal myofibrillar damage during Day 1 (Billingham grade 0), 3 (Billingham grade 0), 5 (Billingham grade 1), and 7 (Billingham grade 1) of culture, and TUNEL staining showed minimal TUNEL+ apoptotic cells. Red arrows: TUNEL+ nuclei. C. Quantification shows a low baseline of TUNEL+ nuclei which did not increase during 7 days in culture (n = 3 per condition). D. Adult mouse cardiac slices had much lower viability when cultured in the same canine media (CM) compared to the Claycomb media (MTT purple staining indicates viable tissue), E. in which tissues showed no significant decrease in viability within the 7 days of culture by MTT staining. F. However, TUNEL staining showed significant increase in apoptotic cells by day 3 of culture. N = 4, ANOVA with Dunnett’s post-test. *p<0.05, **p<0.01, ns = no significant change.

The duration of cell viability within canine and mouse cardiac slices was first quantified in culture by MTT. Cultured canine slices maintained consistently high viability for 7 days, and by day 14, cardiac tissue remained on average 82.4% viable compared to the day of collection (n = 7, p = 0.99 day 14 vs. day 1) (Fig 1A). Histological staining throughout the first 7 days of culture showed minimal myofibrillar damage based on the Billingham pathologic grade [29], and no change in TUNEL staining as a measure of cell death (Fig 1B and 1C). By comparison, the mouse slices were not viable immediately after slicing when cultured in the same media. They were, however, able to maintain viability in the Claycomb media in a 2% CO_2_ environment. Despite no significant change in tissue viability by MTT assay in the Claycomb media through the first 7 days of culture (n = 4, p = 0.54 day 7 vs. day 1), a significant increase in TUNEL+ staining was noted after 3 days of culture, suggesting increase in apoptotic cell death (p = 0.006, day 3 vs. day 1) (Fig 1D–1F). These results also suggest that TUNEL staining analysis is more sensitive to changes in cellular viability than MTT assay. Based on these data, the canine slice model was deemed more suitable for long term culture than mouse slices, and hence all subsequent studies were performed using canine cardiac slices to study autophagic flux.

### Characterization of basal autophagy in canine cardiac slices

**Fig 2:**
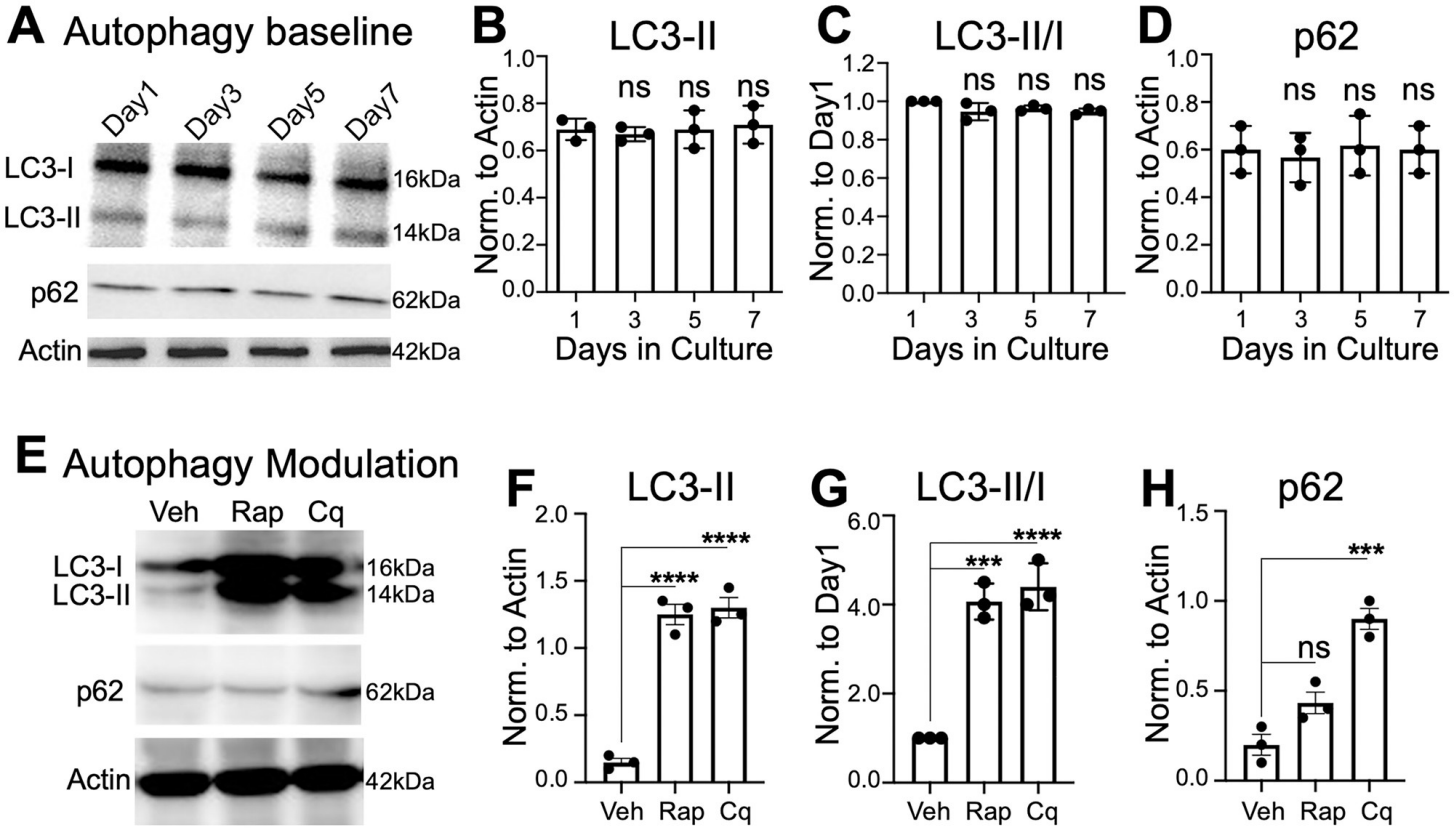
Basal autophagy is preserved in cultured canine cardiac slices. A-D. Representative immunoblots for cardiac slices cultured for 1–7 days. Quantification showed no change in expression of autophagy proteins LC3-II and p62. E-H. Autophagy was induced by 0.1 μM rapamycin (Rap) or blocked by 20 μM chloroquine (Cq) for 24 hours. Rapamycin significantly increased LC3-II expression but not p62 compared to vehicle (Veh) control. Chloroquine led to the accumulation of LC3-II and p62. Protein quantifications were normalized to Actin. N = 3, ***p<0.001, ****p<0.0001, ns = no significant change, ANOVA with Tukey post-test.

Autophagic flux was quantified by measuring the protein levels of autophagy markers LC3-II and p62 in the cardiac slices over the course of culture or post treatment with well-established autophagy modulators (S2 Fig) [14, 30]. Expression levels of these proteins did not significantly change during the 7 days of culture (Fig 2A–2D). Rapamycin inhibits mTOR complex, triggering downstream signaling cascades that upregulate autophagic flux, evidenced by increased LC3 lipidation to form LC3-II (p<0.0001) without impacting the level of p62 (Fig 2E–2H), consistent with autophagy induction that had reached equilibrium [13, 14]. Conversely, autophagy inhibition with chloroquine, which disrupts lysosomal degradation of autophagosomes, led to an accumulation of both LC3-II (p<0.0001) and p62 (p = 0.0004), consistent with autophagic flux blockade. These data support the notion that canine cardiac slices maintain autophagic flux during culture and that the impact of known activator/inhibitor of autophagy can be accurately quantified in these tissue slices.

### Doxorubicin induces apoptotic cell death and impairs autophagy in canine cardiac slices

**Fig 3:**
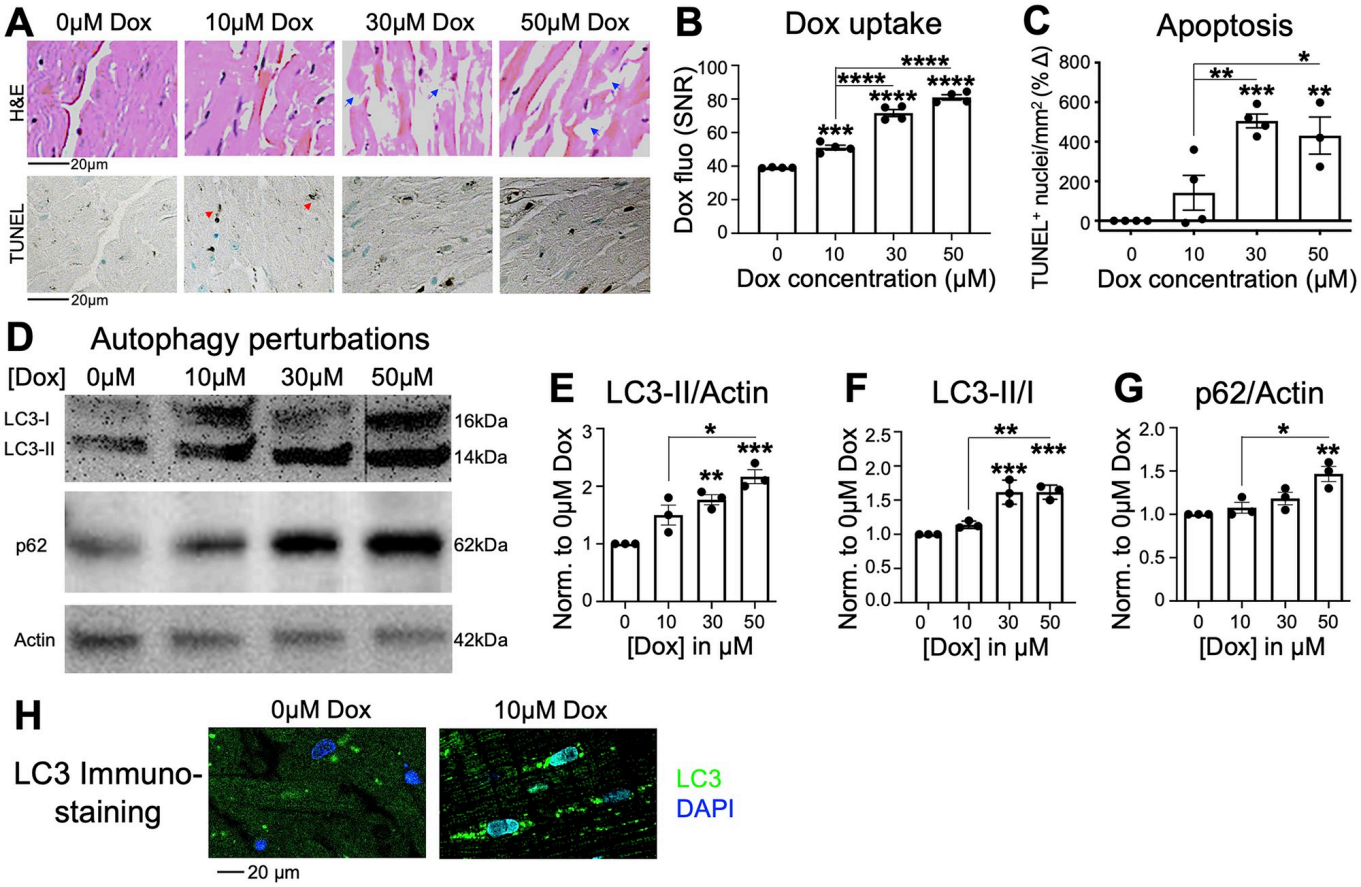
Doxorubicin induces cardiac tissue damage, apoptotic cell death, and impairs autophagy. Canine cardiac slices were exposed to increasing concentrations of doxorubicin (Dox, 10–50 μM) for 24 hours. A. Representative H&E and TUNEL stained sections exposed to 0–50 μM of doxorubicin. 0 μM: Bellingham grade 0; 10 μM: Bellingham grade 2; 30 and 50 μM: Bellingham grade 3. Blue arrows: disorganized myocyte fibers. Red arrows: TUNEL+ nuclei. B. Quantification of doxorubicin retention in cardiac slices by direct fluorescence tissue imaging. SNR = signal-to-noise ratio. N = 4. C. Quantification of TUNEL+ nuclei in cardiac slices exposed to 0, 10, 30, or 50 μM of doxorubicin for 24 hours (n = 4). D-G. Representative western blots and quantification of autophagy proteins (LC3-II, p62) in slices treated with increasing concentrations of doxorubicin for 24 hours. N = 3. H. Immunostaining of LC3 demonstrated robustly increased LC3 accumulation (green = LC3, blue = DAPI) in cardiomyocytes of slices exposed to doxorubicin. *p<0.05, **p<0.01, ***p<0.001, ****p<0.0001 compared to untreated controls unless otherwise noted, ANOVA with Tukey post-test.

Next, we evaluated whether cardiac tissue damage and changes in autophagic flux secondary to doxorubicin treatment could be modeled using canine cardiac slices. We tested increasing doses of doxorubicin between 10–50 μM and quantified the impact on apoptotic cell death and disruption in autophagic flux. Higher doses of doxorubicin were tested to definitively perturbed the autophagic flux. When incubated with doxorubicin for 24 hours, a dose-dependent increase in tissue damage was observed in the H&E and TUNEL stained sections, as evidenced by mild cardiomyocyte disorganization at 10 μM (Bellingham grade 2) and moderate disorganization with 30 and 50 μM (Bellingham grade 3) (Fig 3A). This correlated with the dose-dependent retention of doxorubicin in the cardiac tissue measured by the inherent fluorescence signature of doxorubicin (Fig 3B). To further quantify apoptosis, slices exposed to 10 μM doxorubicin exhibited an average 141.7% increase in TUNEL+ nuclei compared to untreated slices, which increased significantly to 504.7% (p = 0.0005) at 30 μM, and 430.7% (p = 0.0032) at 50 μM (Fig 3C). Concomitant with the rise in TUNEL+ nuclei, doxorubicin treatment also caused autophagy blockade in the cardiac slices. Specifically, there was a dose-dependent accumulation of LC3-II and p62 (Fig 3D–3G and S3 Fig). Consistent with the blockade of autophagic flux, immunostaining showed increased LC3 accumulation in doxorubicin-treated slices compared to untreated slices (Fig 3H and S4 Fig).

### Rapamycin rescued cardiomyocyte cell death by restoring autophagic flux

**Fig 4:**
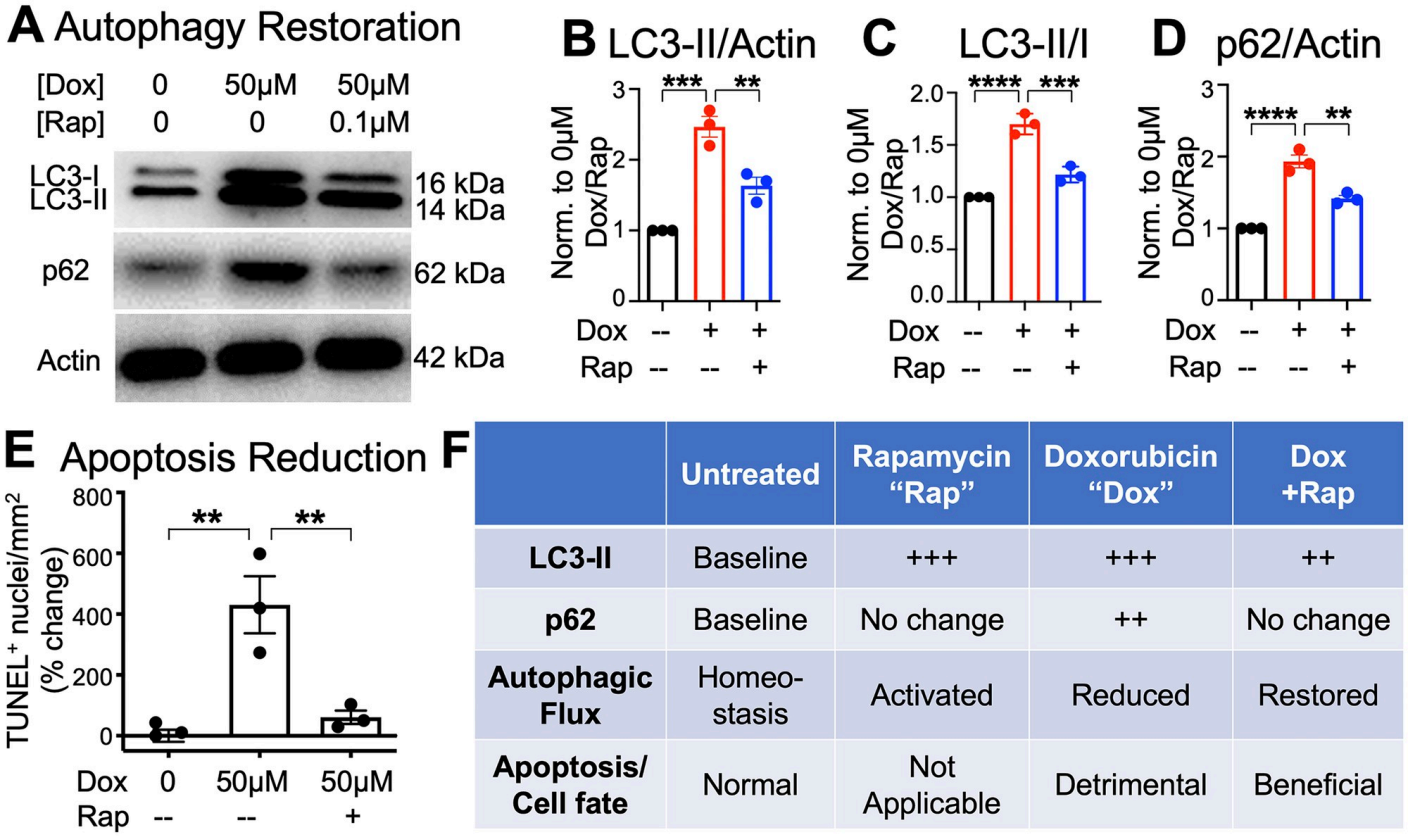
Autophagy induction by rapamycin significantly attenuated cardiomyocyte apoptotic cell death. Cardiac slices were exposed to 50 μM doxorubicin with or without rapamycin (Rap) co-treatment. A-D. Representative immunoblots and quantification of LC3-II and p62. E. Expression increases of LC3-II and p62 with doxorubicin were mitigated with rapamycin co-treatment for 24 hours, leading to cardiomyocyte apoptosis reduction. N = 3. F. The effects of doxorubicin, rapamycin, and chloroquine treatment on LC3-II and p62 expression, and the overall effect on autophagic flux and apoptotic cell death are summarized. **p<0.01, ***p<0.001, ****p<0.0001, ANOVA with Tukey post-test.

To test whether the canine slice model could be used to screen for potential cardioprotective strategies that reverse the detrimental impact of doxorubicin, we investigated the effects of rapamycin co-treatment with doxorubicin on autophagic flux and apoptosis. Rapamycin significantly reduced the accumulation of LC3-II (p = 0.0040) and p62 levels (p = 0.0016) due to doxorubicin treatment (Fig 4A–4D). This was associated with a significant reduction in apoptotic cell death in rapamycin treated cardiac slices compared to treatment with 50 μM doxorubicin alone (p = 0.0045) (Fig 4E and S5 Fig). Effects on the autophagic flux and the impact on apoptotic cell death are summarized in Fig 4F.

## Discussion

We report here the use of adult canine hearts from organ donors to generate a LV cardiac tissue culture model relevant for dissecting the mechanisms and identifying protective strategies secondary to toxin-induced cardiac damage. Specifically, we demonstrate that canine cardiac slices: 1) remain viable in culture for at least a week without induction of apoptosis; 2) maintain autophagic flux for at least 7 days in culture and respond as expected to autophagic flux modulators; 3) develop loss of structural integrity, impaired autophagic flux, and increased apoptosis in response to the cardiotoxic doxorubicin; 4) and can be rescued from doxorubicin-induced toxicity by co-treatment with rapamycin, an autophagy activator. This improved cardiac tissue culture technique allows for the study of the autophagic flux in intact adult cardiac tissue without subjecting the tissue to enzymatic digestion. Importantly, this translational model has the potential to generate critical insights into acute toxic responses to drugs and their associate cellular and molecular effects with the cardiac cells in the context of their native structure, as well as to evaluate the impact of novel treatment strategies designed to prevent cardiac toxicities.

In contrast to the canine cardiac slices, mouse cardiac slices could not be maintained in culture under the same conditions. Despite improved tissue viability with modifications of the culture parameters including the use of Claycomb media with 2% CO_2_, increased apoptotic cell death was still noted after just 3 days. Not only do canine cardiac tissues remain viable in culture for longer periods of time, canine cardiomyocytes possess cellular and molecular features that are more similar to human cardiomyocytes than those from mice. Thus, data generated from canine tissue slices are more likely to have translational relevance for human disease. Furthermore, given the shorter life expectancy of canines compared to humans, especially after cancer diagnosis, longitudinal studies involving terminal tissue harvest can be accomplished within 1–2 years, as opposed to decades required for similar human studies. Consequently, the impact of cardiotoxic treatments can be readily assessed in the context of treating canine patients with spontaneous cancer, permitting interactions among co-morbidities, cancer specific factors and drugs to be studied in this model system [26, 31–34].

Our data further demonstrated that the canine cardiac slice culture system can be used to study the effects of cancer treatment on the autophagic process. The slices showed stable basal levels of autophagy and responded appropriately to rapamycin, an activator of autophagic flux, and chloroquine, an inhibitor that blocks autophagosome and lysosome fusion. Furthermore, we noted that autophagic flux blockade by doxorubicin led to cardiomyocyte cell death, similar to previous reports using isolated murine and human cardiomyocytes and murine hearts *in vivo* [16, 18]. It is worth noting that the canine cardiac slices exhibited LC3-II accumulation and increased p62 expression, indicative of acute autophagy blockade [13], in contrast to the autophagy precursor-limited flux blockade previously reported to occur in human embryonic stem cell-induced cardiomyocytes where p62 was noted to decrease in conjunction with an increase in LC3 [18]. This acute impairment in autophagic flux in the canine cardiac slices can be restored by rapamycin co-treatment, mitigating cardiac cell death induced by doxorubicin.

While boosting autophagy prior to doxorubicin administration can be cardioprotective, excessive initiation of autophagy after doxorubicin exposure may be detrimental [35]. Doxorubicin exposure for 24 hours induced cell death in canine cardiac slices and is associated with autophagic flux blockade, evident by the accumulation of autophagy proteins (LC3-II, p62) and the increase in apoptosis. This is consistent with previous reports in neonatal rat cardiomyocytes and mouse hearts, supporting the pathophysiological role of cardiac autophagy in doxorubicin-induced toxicity, where blockade of autophagic flux and accumulation of autophagosomes exacerbates cell death [18, 36, 37]. Our results confirm that the doxorubicin-induced autophagic blockage phenomenon also occurs in canine adult intact heart tissues and in a species regularly treated with doxorubicin for cancer therapy. As such, the canine cardiac culture system provides a platform to investigate effects of autophagic blockage in heart tissue acquired from actual canine patients treated with doxorubicin.

Current clinical options to prevent chemotherapy-induced cardiotoxicity are limited and not routinely used [38]. Autophagy modulation achievable by a myriad of FDA-approved drugs represents a viable cardioprotective strategy, but approaches exploiting this biology have not yet been translated into the clinical setting [39, 40]. While the cardiotoxic effects of doxorubicin are attributed predominantly to direct cardiomyocyte injury, newer targeted therapies such as tyrosine kinase inhibitors may act through vascular cell damage and impact cardiomyocyte autophagy, both of which may lead to systolic dysfunction or heart failure [41–43]. The cardiac slice culture addresses such limitations by encompassing the various cell types and tissue architecture, which will allow visualization of protein important in characterizing autophagic flux, such as LC3 and p62, by immunostaining as well as quantification by protein blot in slices while preserving structural and electrophysiological interactions between these cell types [20, 24, 25, 44, 45]. Consequently, the canine cardiac slice model represents a viable platform for screening cancer treatments for cardiotoxicity while also providing a mechanism to screen novel strategies designed to mitigate these toxicities.

### Limitations

Several limitations should be acknowledged. Our *ex vivo* system does not include electrical stimulation of the cardiac tissue, which would replicate even more closely the native state of the cardiomyocytes. Despite this, we were still able to demonstrate stable basal levels of autophagy with normal flow of the autophagic process. Comparison of results with human cardiac slices would also help determine any cross-species differences. However, healthy human donor hearts are difficult to obtain to perform such comparisons. This highlights the advantage of using canine hearts that can be easily obtained in a humane fashion from patients. Because we are utilizing heart tissues from client-owned dogs of various breeds, age group, and may have other systemic diseases not known at the time of tissue collection; for example, many of them were obese, these factors may affect cardiac function. Given our sample size, we cannot make any conclusions regarding how these factors may influence the tissue responses to doxorubicin or rapamycin. Future work will be needed to better understand the effects of these factors on autophagy. Nevertheless, a potential advantage is that these are similar co-morbidities experienced by human patients, allowing such studies to reflect more clinically relevant conditions.

## Conclusion

We report here the validation of adult canine cardiac slice culture as a novel platform relevant for use in drug-induced cardiotoxicity studies, including those designed to elucidate the mechanistic underpinnings of autophagy and its disruption in cancer drug-induced injury. Our data further support testing of autophagy modulation as a protective therapy during cardiotoxic cancer treatment. This *ex vivo* system will enable future studies that incorporate the influence of cancer intrinsic factors on cardiotoxicity with the use of hearts acquired from canine oncology patients at the time of euthanasia.

## Supporting information

S1 Fig Treatment timeline for canine cardiac slices. (TIF)

S2 Fig Diagram of autophagic flux activation and inhibition. Schematic showing the targets of rapamycin and chloroquine in the autophagic pathway. Rapamycin activates autophagy by upregulating LC3-II lipidation. Chloroquine inhibits autophagic flux by inhibiting fusion of lysosomes with autophagosomes thus autophagosome degradation.
(TIF)

S3 Fig Protein blots of cardiac slices after 48 hours of doxorubicin exposure. Canine cardiac slices were exposed to increasing concentrations of doxorubicin (Dox, 10–50 μM) for 48 hours. Representative western blots and quantification of autophagy protein (LC3-II) in slices treated with increasing concentrations of doxorubicin for 48 hours. N = 3. *p<0.05, **p<0.01 compared to untreated controls unless otherwise noted, ANOVA with Tukey post-test.
(TIF)

S4 Fig LC3 immunostaining after 48 hours of doxorubicin exposure. Immunostaining of LC3 demonstrated robustly increased LC3 accumulation (green = LC3, blue = DAPI) in cardiomyocytes of slices exposed to doxorubicin for 48 hours.
(TIF)

S5 Fig TUNEL staining of slices treated with doxorubicin with or without rapamycin. Representative TUNEL stained cardiac slices exposed to 50 μM doxorubicin (Dox) with or without rapamycin (Rap) co-treatment.
(TIF)
