# Supplementary figures and images for: Using cultured canine cardiac slices to model the autophagic flux with doxorubicin
Source: PLoS One. 2023 Mar 16;18(3):e0282859. doi: 10.1371/journal.pone.0282859 (PMC10019679; doi:10.1371/journal.pone.0282859)

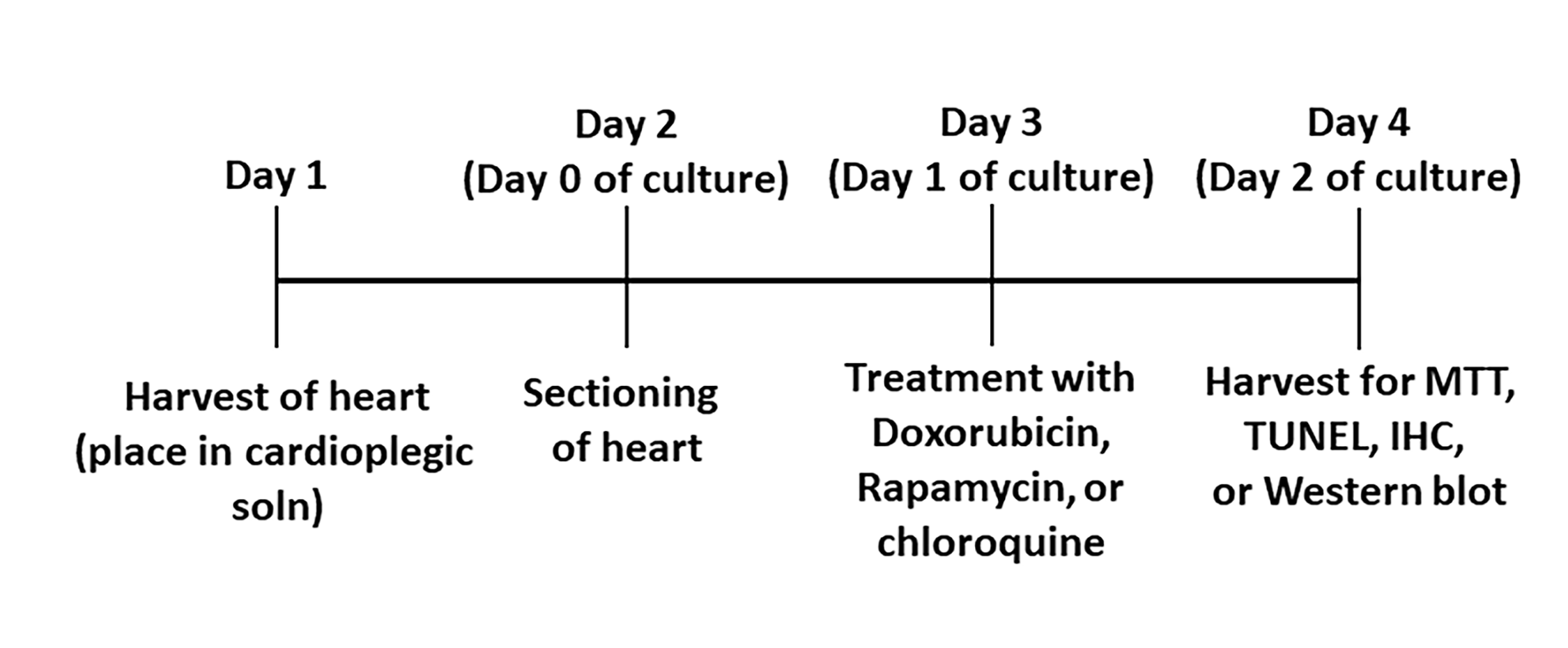

Supplement: S1 Fig — (TIF) [file pone.0282859.s001.tif]

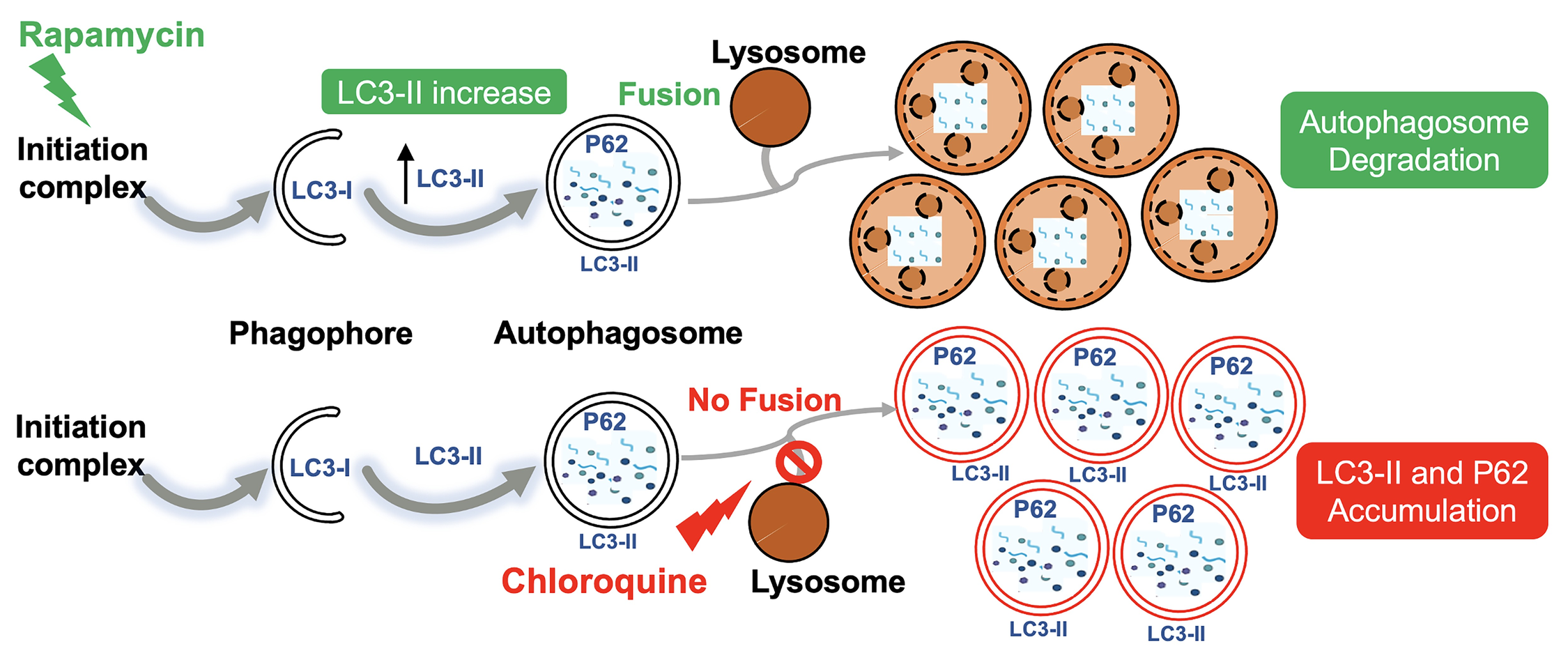

Supplement: S2 Fig — Schematic showing the targets of rapamycin and chloroquine in the autophagic pathway. Rapamycin activates autophagy by upregulating LC3-II lipidation. Chloroquine inhibits autophagic flux by inhibiting fusion of lysosomes with autophagosomes thus autophagosome degradation. (TIF) [file pone.0282859.s002.tif]

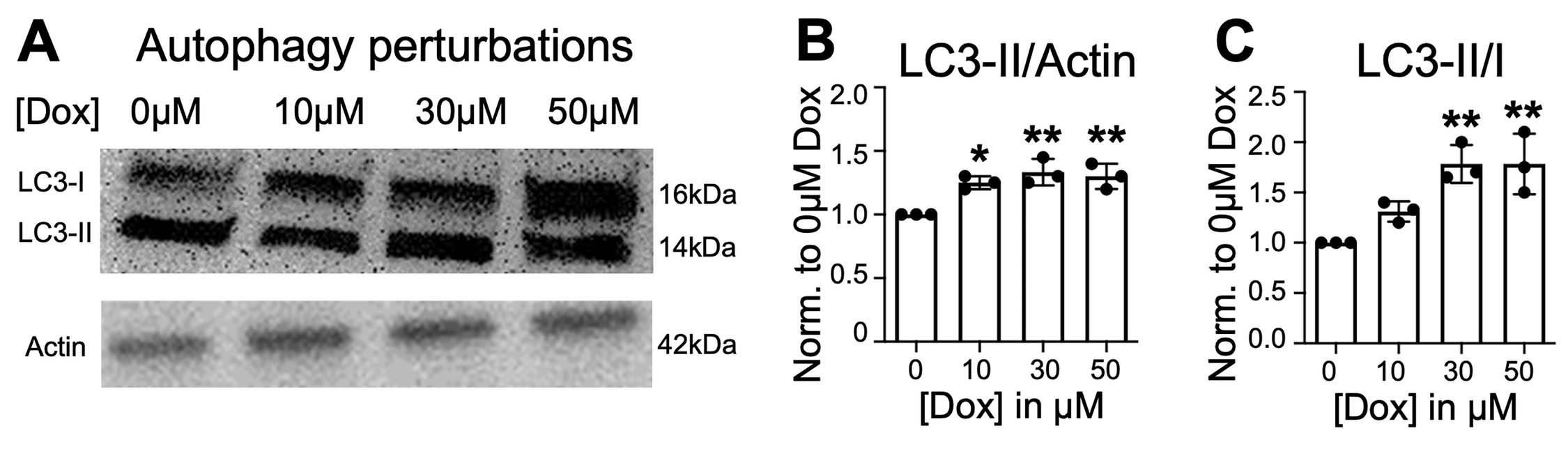

Supplement: S3 Fig — Canine cardiac slices were exposed to increasing concentrations of doxorubicin (Dox, 10–50 μM) for 48 hours. Representative western blots and quantification of autophagy protein (LC3-II) in slices treated with increasing concentrations of doxorubicin for 48 hours. N = 3. *p<0.05, **p<0.01 compared to untreated controls unless otherwise noted, ANOVA with Tukey post-test. (TIF) [file pone.0282859.s003.tif]

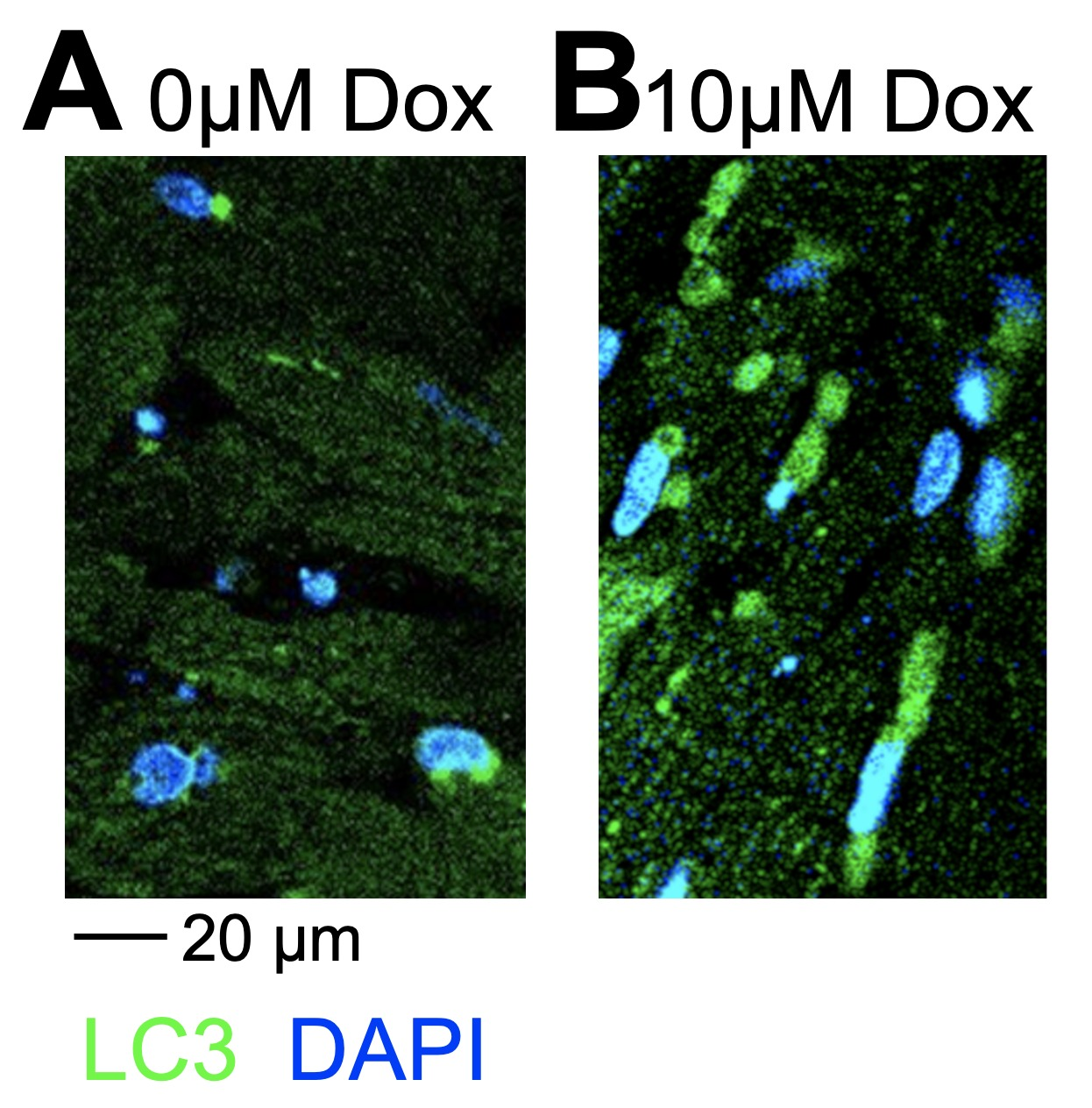

Supplement: S4 Fig — Immunostaining of LC3 demonstrated robustly increased LC3 accumulation (green = LC3, blue = DAPI) in cardiomyocytes of slices exposed to doxorubicin for 48 hours. (TIF) [file pone.0282859.s004.tif]

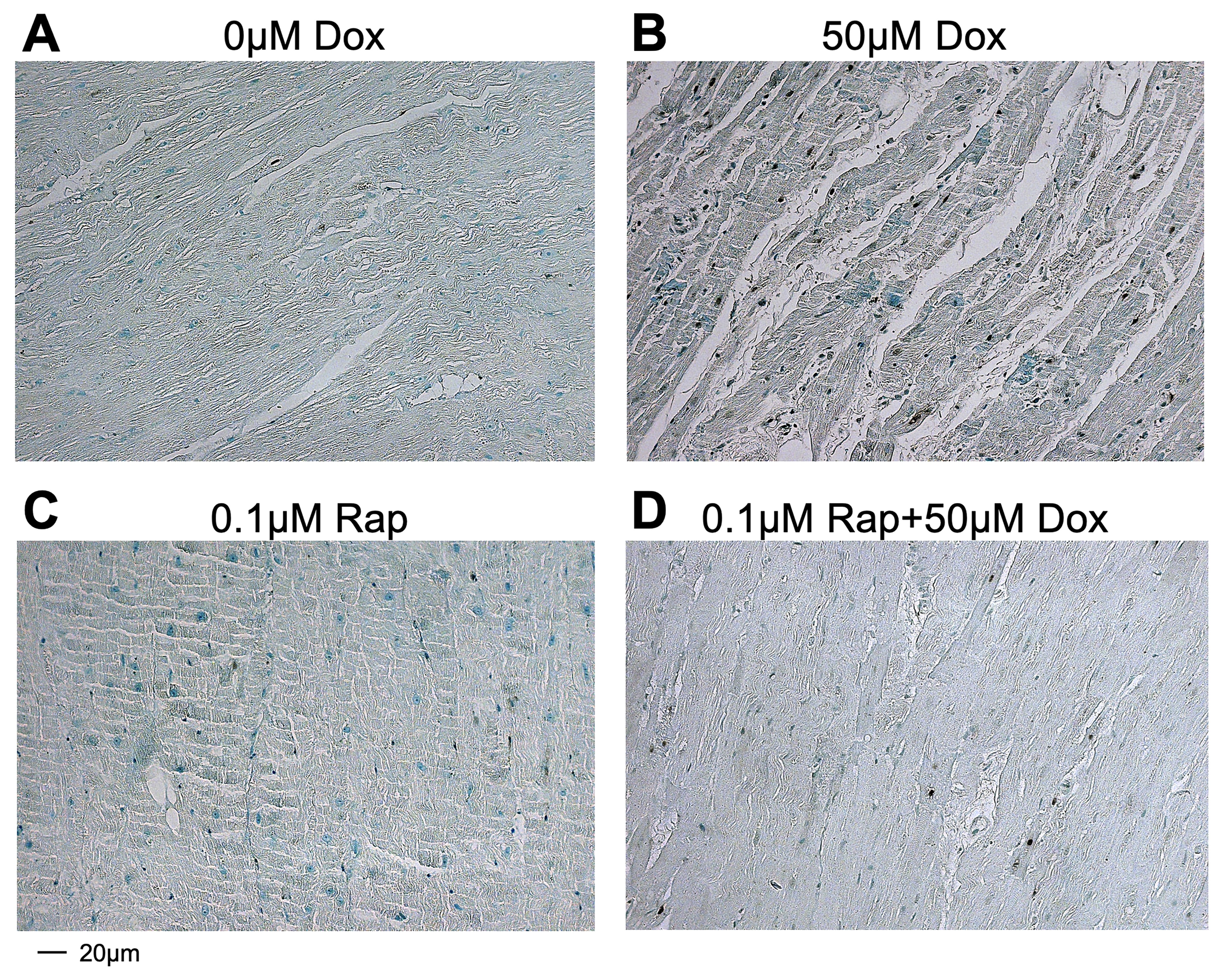

Supplement: S5 Fig — Representative TUNEL stained cardiac slices exposed to 50 μM doxorubicin (Dox) with or without rapamycin (Rap) co-treatment. (TIF) [file pone.0282859.s005.tif]
